# COVID-19 Return to Sport: NFL Injury Prevalence Analysis

**DOI:** 10.2196/35862

**Published:** 2022-04-22

**Authors:** Troy B Puga, Joshua Schafer, Prince N Agbedanu, Kevin Treffer

**Affiliations:** 1 College of Osteopathic Medicine Kansas City University Kansas City, MO United States; 2 School of Medicine University of Kansas Kansas City, KS United States; 3 Department of Health Sciences Division of Science, Technology, Engineering, and Math Friends University Wichita, KS United States; 4 Department of Osteopathic Manipulative Medicine College of Osteopathic Medicine Kansas City University Kansas City, MO United States

**Keywords:** COVID-19, sport, injury, prevalence, cause, data, statistics, pain, training, practice, physiology, adaptation

## Abstract

**Background:**

Sport injuries have been common among athletes across the globe for decades and have the potential to disrupt athletic careers, performance, and psyche. Many health professionals and organizations have undertaken injury mitigation strategies to prevent sport injuries through protective equipment, training protocols, and a host of other evidence-based practices. Many of these specialized training methods were disrupted due to protocols to mitigate the spread of COVID-19. This research examines the effects of the COVID-19 pandemic in relation to the prevalence of athletic injuries in the National Football League (NFL).

**Objective:**

During the COVID-19 pandemic, NFL teams and athletes across all levels of sport were reported to have reduced training in preparation for their seasons due to protocols to mitigate the spread of COVID-19. This study compares the prevalence of injury during the 2018, 2019, and 2020 NFL seasons, with the aim to determine the potential causes of the differences in injury prevalence.

**Methods:**

Official injury reports from each team were counted during the 17-week regular season of each year (2018, 2019, and 2020). The data were analyzed using an unpaired t test to compare the injury prevalence between each of the three seasons.

**Results:**

The 2018 season produced a total of 1561 injuries and a mean of 48.8 injuries per team. The 2019 season produced a total of 1897 injuries and a mean of 59.3 injuries per team, while the 2020 season produced a total of 2484 injuries and a mean of 77.6 injuries per team. An unpaired t test was performed using the data to compare the mean number of injuries per team during each of the seasons. Comparison of the 2020 season against the 2019 season showed a statistically significant difference (*P*<.001); comparison of the 2020 season to the 2018 season found a statistically significant difference (*P*<.001); and comparison between the 2019 and the 2018 seasons found a statistically significant difference (*P*=.03).

**Conclusions:**

Although the 2019 and 2018 seasons showed a statistically significant difference (*P*=.03), this difference is not as large when we compare the 2020 seasons versus the 2019 (*P*<.001) and 2018 (*P*<.001) seasons. The astronomical increase in injury prevalence during the 2020 season over the previous years raises the possibility that there was a reduced physiological adaptation to stress, due to the limited amount of training as a result of the closure of practice facilities in order to slow the spread of COVID-19.

## Introduction

The National Football League (NFL) is a professional American football league composed of 32 teams. The NFL is composed of high-level, elite athletes who are able to train rigorously and consistently at state-of-the-art facilities, with the assistance of some of the best trainers and medical professionals in the world. As the COVID-19 pandemic spread across the world, many professional, collegiate, and amateur sports were brought to a halt [1]. The majority of athletes across all levels of competition across the United States and the rest of the world were unable to compete in organized sports or in-person training activities due to health precautions of COVID-19 [1,2]. In addition, NFL team facilities were closed from March 25, 2020, to May 19, 2020, in order to help mitigate the spread of COVID-19 [3]. Research has reported reductions in training frequency and availability for athletes across the United States during the COVID-19 shutdown period between March and June of 2020 [1]. During home confinement due to the COVID-19 shutdown, athletes likely experienced detraining, which is the loss of previous training-induced physiological adaptations caused by a lack of sufficient training stimulus [4]. It has been shown that detraining has deleterious effects at the level of the muscle. One study showed that just 8 weeks of detraining results in decreased muscle mass, muscle strength, and overall muscle power [5]. It is also postulated that these effects in skeletal muscle could significantly increase the risk of injuries, especially within contact sports [5]. Overall, detraining has been proven to negatively impact an athlete’s physical performance [6]. The last time NFL players saw restricted access to training facilities was when the NFL underwent a lockout in 2011 for 14 weeks. As the 2011 NFL lockout ended and training camp began, there was a marked increase in Achilles tendon ruptures during training camp and preseason [7]. Preseason injuries could not be tracked during the 2020 NFL season, as these games were canceled in order to help reduce the spread of COVID-19.

American football is a contact sport, with at least some level of contact occurring on every play. Given that American football is a contact sport, it comes with nonmodifiable risks of injury [8]. In order to reduce the risk of injury, athletes prepare their bodies through vigorous training and strict dietary control so that they can stay healthy for as long as possible. Though no amount of training can completely exclude an athlete from getting injured, physiologic bone remodeling after high-intensity workouts offer some amount of protection from the extreme impact forces placed on the players’ bodies during competition [9]. The Wolff law states that bones will adapt to the degree of mechanical loading, such that an increase in loading will cause the architecture of the internal and external bone layers to become stronger [9,10]. Conversely, a decrease in loading will cause a decrease in bone strength [9,10]. The duration, magnitude, and rate of force applied to the bone dictate the way in which the integrity of the bone is subsequently altered [9,10].

Resistance exercise is a method of conditioning in which an individual works against resistive loads such as free weights, resistance bands, or body weight in order to increase sport performance or overall health and strength [11]. Resistance exercise has been shown to create a significant acute hormonal response, which is important for tissue growth and remodeling [12]. Anabolic hormones have been shown to elevate 15 to 30 minutes after resistance exercise, providing an adequate stimulus [12]. These anabolic hormones are known to be crucial for skeletal muscle resistance training adaptations [13]. Exercise adaptations lead to physiological changes in muscles and tendons that can be advantageous to improving athletic performance, such as hyperplasia or hypertrophy [14,15]. Resistance training has also demonstrated changes in body composition, neuroendocrine function, and cardiovascular response to stress [16].

In this study, we aimed to determine if there is a change in injury prevalence during the COVID-19 season (2020). We hypothesize that there will be an increase in overall number of injuries and an increase in the mean number of injuries per team during the COVID-19 season (2020). If true, we further hypothesize that this increase in injuries may be due to reduced physiological adaptations of training due to lack of access to sufficient training facilities and training stimuli, stemming from COVID-19 health precautions.

## Methods

### Study Design

The number of injuries for each team was tallied during the 17-week long NFL regular season using the weekly medical data injury reports that are published publicly by each team. If an official team report was not available through the individual team, deferment was made to the injury report on the official NFL website. Athletes on the injury reports for the same injury for consecutive weeks were only counted once; however, athletes were counted again if they presented with a new injury to a different anatomical region. Illnesses, COVID-19–positive cases, holidays, and nonmedical days off were not included in the total tally. Illnesses were not included in the final tally because they are not within the scope of this research. An injury has been defined as a physical complaint during a match or training that affects performance; therefore, illnesses should be reported separately from the incidence of physical complaints [17]. Because football is a contact sport, contact injuries were included in the study, as contact is a nonmodifiable risk factor for injury [8]. The total tallies of injuries per team were compared to those of the previous season and statistically analyzed using an unpaired *t* test.

### Data Analysis

**Figure 1:**
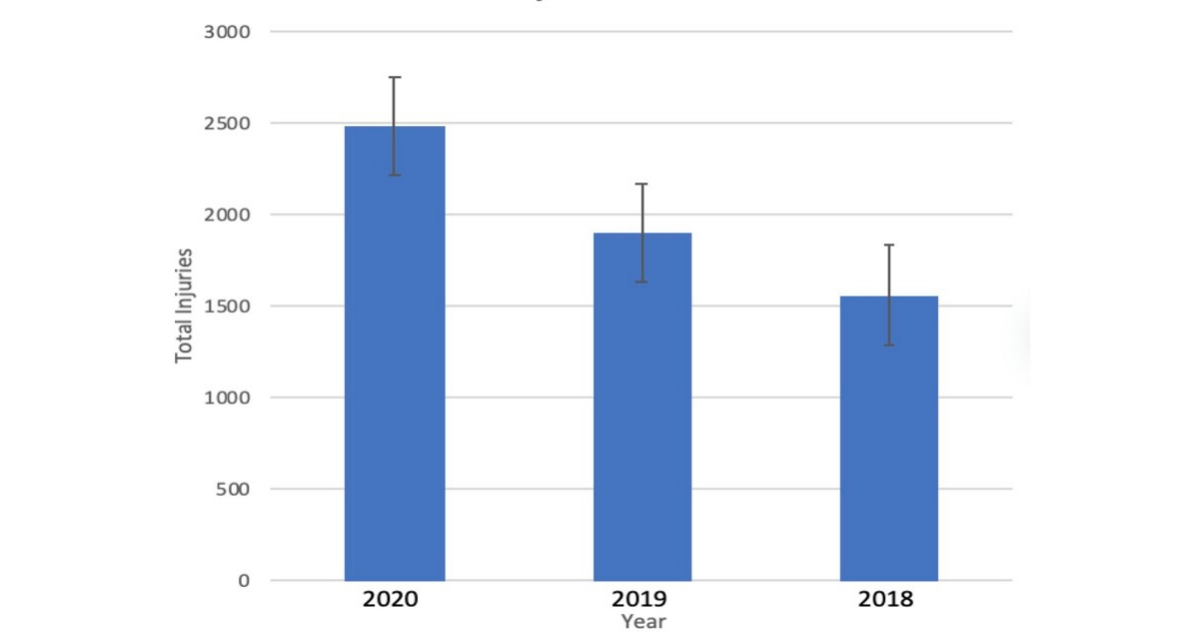
Total injuries per National Football League season.

A data analysis was conducted by comparing the three different seasons. An unpaired *t* test was performed on the data set to compare the mean number of injuries per team per season to each of the three seasons. Figure 1 illustrates total injuries per NFL season.

## Results

**Figure 2:**
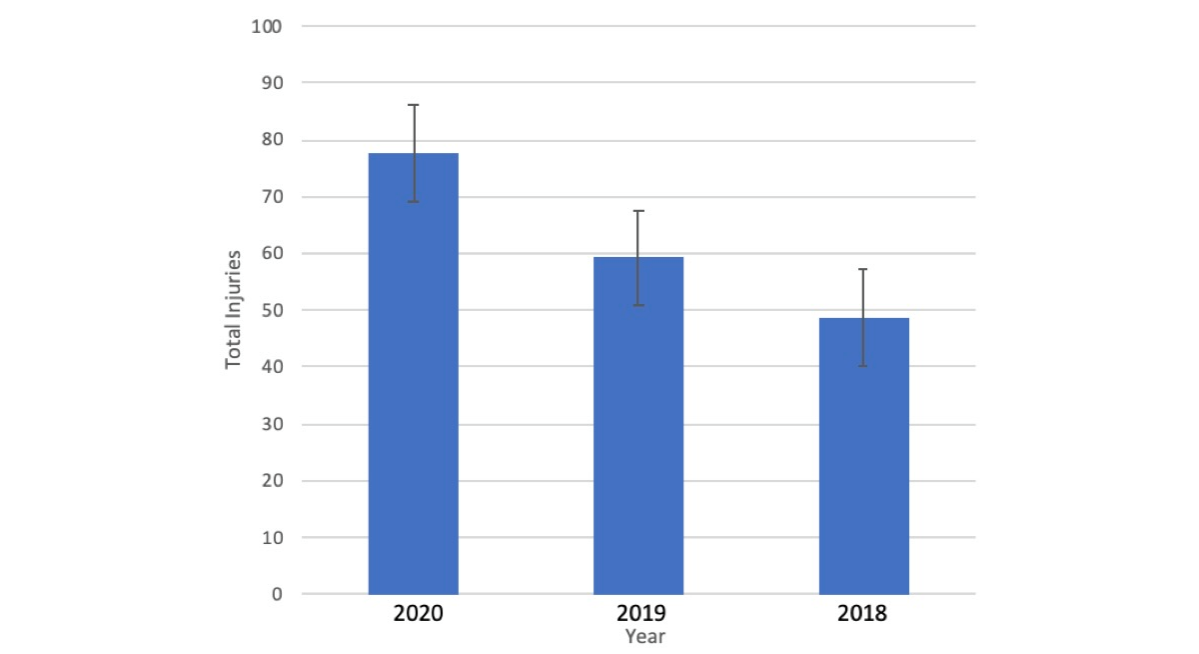
Mean injuries per team per National Football League season.

After tallying the injuries, the 2020 season produced a total of 2484 injuries with a mean number of 77.6 injuries per team. The 2019 season produced a total of 1897 injuries with a mean number of 59.3 injuries per team. The 2018 season produced 1561 injuries with a mean number of 48.8 injuries per team. An unpaired *t* test was performed to compare the mean number of injuries per team of each season. Comparison of the 2020 season against the 2019 season showed a statistically significant difference (*P*<.001). Comparison of the 2020 season to the 2018 season also showed a statistically significant difference (*P*<.001). Comparison between the 2019 and the 2018 seasons showed a statistically significant difference (*P*=.03) as well. Figure 2 shows mean injuries per team per NFL season.

## Discussion

The results of this study demonstrated an increased number of overall injuries in the 2019 season and 2020 season when compared to the 2018 season. The results of the study also demonstrated an increased mean number of injuries per team during the 2019 season (*P*=.03) and the 2020 season (*P*<.001) when compared to the 2018 season. The 2020 season also demonstrated a statistically significant (*P*<.001) increase in the mean number of injuries per team when compared to the 2019 season. There could be several possible factors that play into the injury prevalence between the three NFL seasons. However, we believe that a decrease in physiological adaptation, due to reduced access to training facilities during the COVID-19 pandemic, contributed to the difference in injury prevalence during the 2020 NFL season when compared to the 2018 and 2019 seasons.

We acknowledge that there may be limitations to the study in that it may not have accounted for other injuries, such as injuries sustained during the preseason of the 2018 and 2019 seasons, injured reserve players, and unreported injuries. We also admit that there is a limitation due to an inability to calculate the exact hours of training per season. This calculation would be significantly hindered, as we used a public data set and do not have access to the individuals’ training regimens within the data set. Furthermore, even if training hours could be gathered, there would be a potential for significant recall bias in any survey that attempted to calculate these hours, as the data go several years into the past. While we admit this is a limitation, we have created the best possible scenario by showing there was closure of the NFL training facilities between March 25, 2020, and May 19, 2020 [3], and demonstrating decreased training during the COVID-19 lockdown period in the United States among the majority of athletes and across all levels of sport [1].

Training is important for athletes as it can induce advantageous physiological adaptations to bone, muscles, and tendons when provided with an adequate stimulus [9-15]. Training positively impacts athletic performance, while detraining has shown to negatively affect athletic performance [5,6]. With all of the information regarding facility closures, reduced training during the COVID-19 pandemic, and the effects of training and detraining on athletes, we can confidently conclude that decreased physiological adaptations secondary to a reduction in training contributed to the increase in overall injuries and mean number of injuries during the 2020 NFL season. Further support of this conclusion is provided by the past NFL shutdown in 2011, which showed an increase in Achilles tendon ruptures among players during the preseason [7].

The demonstration of increased injury prevalence during the 2020 NFL season could be impactful to athletic programs around the world and allow organizations to focus on strategies to mitigate injuries in the future. This information may also be useful for understanding sport preparation and rehabilitation protocols. There should be more work carried out, at both the professional and amateur levels, to determine the training intensity necessary for physiological adaptation to occur that will reduce injuries. There must also be further follow-up to see if there was also a rise in injuries at the collegiate and amateur levels of sport.

Potential follow-up studies could include analyzing the prevalence of injuries for individual teams with severe COVID-19 outbreaks, examining the relationship between geographic COVID-19 hotspots and number of athletic injuries, and examining injury prevalence at the collegiate and amateur levels of sport. We invite other researchers to continue researching the number of injuries in athletes before and after the COVID-19 lockdown measures.

## Abbreviations

NFL: National Football League
